# The Effect of a Personalized Automated Exercise Guidance System on the Improvement of obesity

**DOI:** 10.1093/geroni/igaa057.3093

**Published:** 2020-12-16

**Authors:** Yeonsik Noh, Jae-Seung Chang, Ki Chon, Hyungro Yoon, In Cheol Jeong, In Deok Kong

**Affiliations:** 1 University of Massachusetts Amherst, Amherst, Massachusetts, United States; 2 Yonsei University Wonju College of Medicine, Wonju, Republic of Korea; 3 University of Connecticut, Storrs, Connecticut, United States; 4 Icahn School of Medicine at Mount Sinai, New York, New York, United States

## Abstract

This study introduces a personalized automated exercise guidance system for the efficient reduction of obesity. The proposed system was composed of wearable biometric devices and exercise-machine control systems connected with the integrated database server. It was designed for providing customized exercise prescription (intensity, repetition, frequency, etc.) according to ACSM guidelines for obesity based on real-time biosignals feedback and an individual’s exercise capacity. Nineteen subjects participated in evaluating the proposed system, and we found that the risk factors (body composition, hemodynamics, blood enzymes, and exercise variables) related to obesity were statistically significantly improved. Other exercise prescriptions for chronic diseases and symptoms will be able to adapt to the proposed system so that we believe it would be substantially helpful to improve geriatric diseases and symptoms by using technology-driven exercise.

